# A rapidly progressive foot drop caused by the posttraumatic Intraneural ganglion cyst of the deep peroneal nerve

**DOI:** 10.1186/s12891-018-2229-x

**Published:** 2018-08-18

**Authors:** Hui Lu, LiFeng Chen, Shuai Jiang, Hui Shen

**Affiliations:** 1 Department of Hand Surgery, The First Affiliated Hospital, College of Medcine, ZheJiang University, 79# Qingchun Road, HangZhou, ZheJiang Province, 310003 People’s Republic of China PR; 2 Department of Medical Engineering, The First Affiliated Hospital, Zhejiang University, #79 Qingchun Road, Hangzhou, Zhejiang Province, 310003 People’s Republic of China

**Keywords:** Intraneural ganglion cyst, Foot drop, Deep peroneal nerve

## Abstract

**Background:**

Intraneural ganglion cysts usually arise from the articular branch of the nerve. The relationship between intraneural ganglion cysts and trauma is not clear.

**Case presentation:**

We report a case of a 62-year-old female with a rapidly progressive foot drop caused by a posttraumatic intraneural ganglion cyst of the deep peroneal nerve. We excised the ganglion cyst and performed nerve decompression. After the surgery, the patient had a functional recovery.

**Conclusions:**

The concurrence of an intraneural ganglion cyst and trauma may increase damage to the nerve, although it is difficult to diagnosis before an operation. Early diagnosis and early proactive interventions would likely be associated with a good outcome.

## Background

Intraneural ganglion cysts are rare benign lesions around the peripheral nerves near joints or tendon sheaths that are located within the perineurium or epineurium. The common peroneal nerve is the most frequently involved nerve, although cysts at the ulnar, sciatic, and tibial nerves have also been reported [1–3]. Intraneural ganglion cysts commonly result in pain, numbness, or paralysis. Complete motor dysfunction is relatively rare. The etiology of intraneural ganglion cysts is not clear. Articular unification is the most widely accepted cause, because cysts usually attach to the synovial joint along the nerve articular branch [4].

We report a case of an intraneural ganglion cyst of the deep peroneal nerve. The lesion was located at the level of the proximal fibular head, leading to foot drop after trauma. The rapid progression has not been reported in the literature. We will discuss the relationship of the intraneural ganglion and trauma in this report.

## Case presentation

**Fig. 1:**
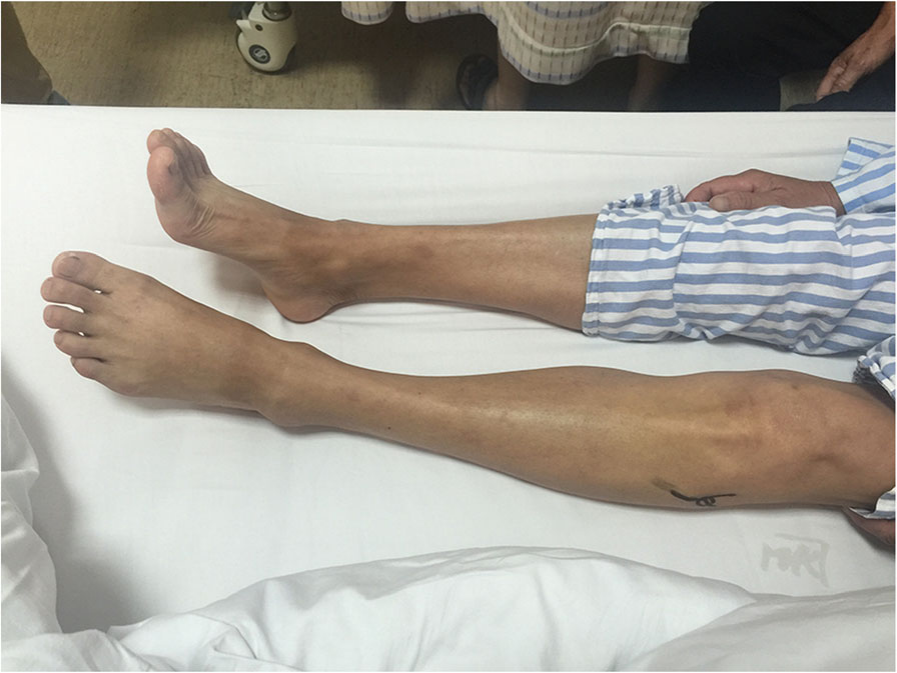
Complete foot drop and ecchymoses were seen on the left lateral of knee and calf

**Fig. 2:**
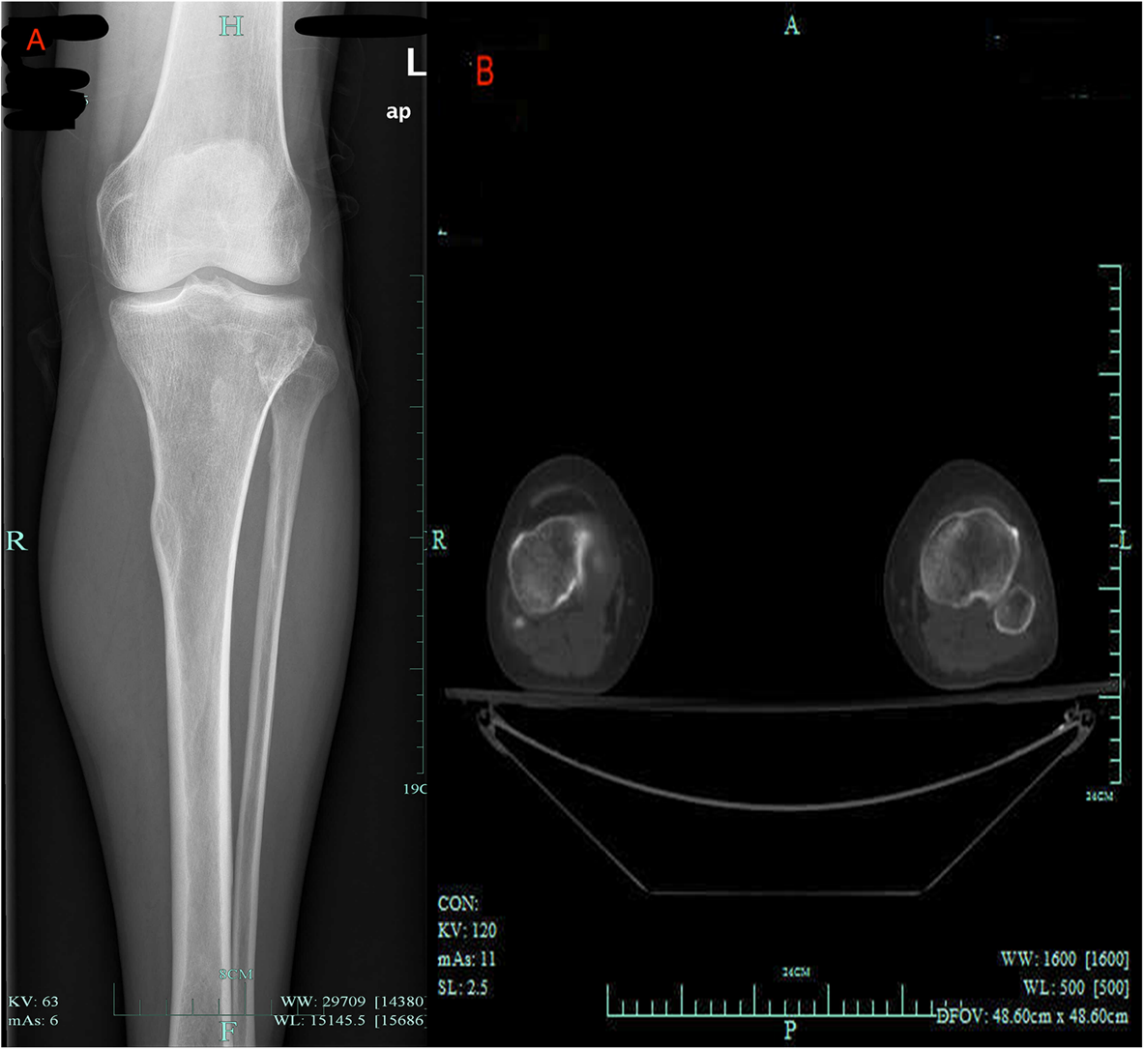
A X-ray documented no fracture. B Computed tomography (CT) documented no fracture

A 62-year-old female patient presented at our hospital with pain in the left lateral keen and an acute foot drop. She had had a traffic accident 12 days before and received neurotrophic drug treatment (Methylcobalamin 1500 μg, intramuscular, daily). The patient had no history of lumbar disc disease. Physical examination showed complete foot dorsiflexion in the left ankle. Neurologic examination revealed numbness on the contiguous side of the first and second toes. Tinel’s sign was positive at the level of the proximal fibular head. Ankle dorsiflexion and large toe extension showed severe weakness (Medical Research Council (MRC) rating scale grade 2). Ecchymoses were seen in the left lateral of knee and calf (Fig. 1). She did not receive magnetic resonance imaging (MRI) because of her cardiac pacemaker and was not examined by ultrasound because no superficial mass was detected. Electromyography (EMG) showed a deep left peroneal nerve axonal neuropathy, decreased nerve conduction velocity, motor amplitude, and denervation potential in the extensor hallucis longus (EHL) and tibialis anterior muscle (TA) (Table 1). X-ray and computed tomography (CT) revealed no fractures (Fig 2). Laboratory studies revealed routine blood test results and tumor markers, and erythrocyte sedimentation rate (ESR) and high-sensitivity C-reactive protein was within the normal range.

**Table 1:** The Preoperative Electromyography of patient

Motor nerve conduction				
Nerve and Site	incubation	amplitude	speed	area
Peronel.L				
Fibila(head)	5.3 ms	0.4 mV	37.6 m/s	1.5mVms
Popliteal fossa	12.8 ms	0.3 mV	m/s	1.3 mVms
TAM	2.7 ms	3.0 mV	m/s	17.9mVms
Tibial.R				
Fibula(head)	5.6 ms	21.3 mV	m/s	56.4 mVms
Popliteal fossa	15.0 ms	20.4 mV	41.5 m/s	60.8 mVms
Peroneal.R				
Fibula(head)	4.0 ms	5.8 mV	41.8 m/s	23.7mVms
Popliteal fossa	11.4 ms	5.1 mV	m/s	21.9mVms
Tibial.L				
Fibula(head)	6.7 ms	20.8 mV	m/s	49.0 mVms
Popliteal fossa	16.1 ms	16.0 mV	40.8 m/s	42.4 mVms
F wave				
	M Wave Latency	F Wave Latency	F-M wave interval	F Wave Occurrence rate
Tibial.L	6.1	55.3	49.2	100
Sensory nerve conduction				
Nerve and Site	incubation	amplitude	speed	
Sural.L				
Fibula(head)	3.4 ms	7.2 μV	38.8 m/s	
Superficial peroneal.L				
Fibula(head)	2.7 ms	14.7 μV	47.7 m/s	
Sural.R				
Fibula(head)	2.5 ms	18.0 μV	46.7 m/s	
Superficial peroneal.R				
Fibula(head)	2.1 ms	20.9 μV	49.5 m/s	

*EMG* Electromyography showed a left deep peroneal nerve axonal neuropathy, decreased nerve conduction velocity, and motor amplitude and denervation potential in the *EHL* extensor hallucis longus and *TA* tibialis anterior muscles

**Fig. 3:**
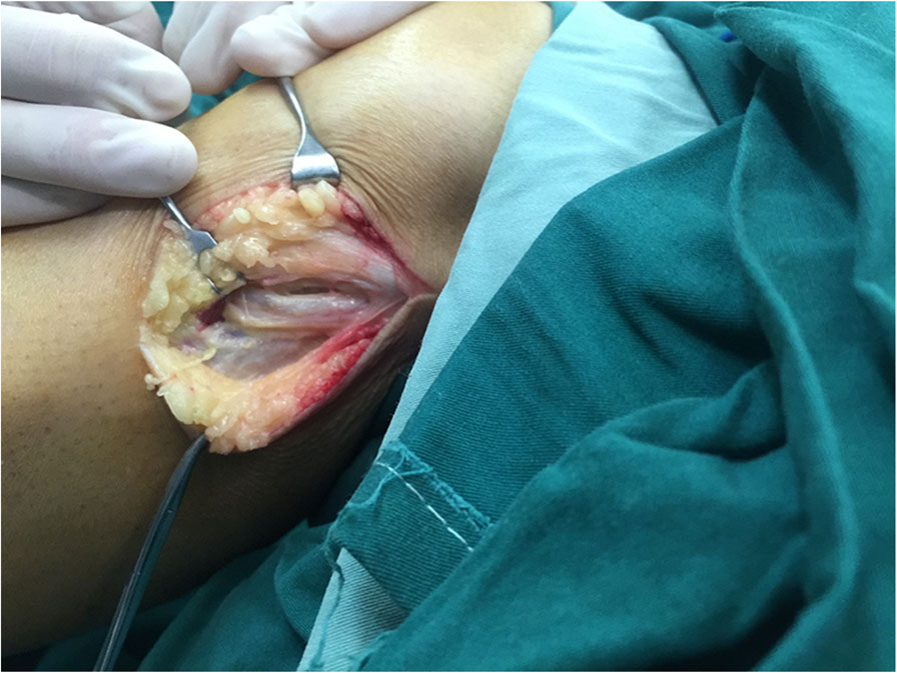
The epineurium of common fibular nerve was a scar proliferation adhesion

**Fig. 4:**
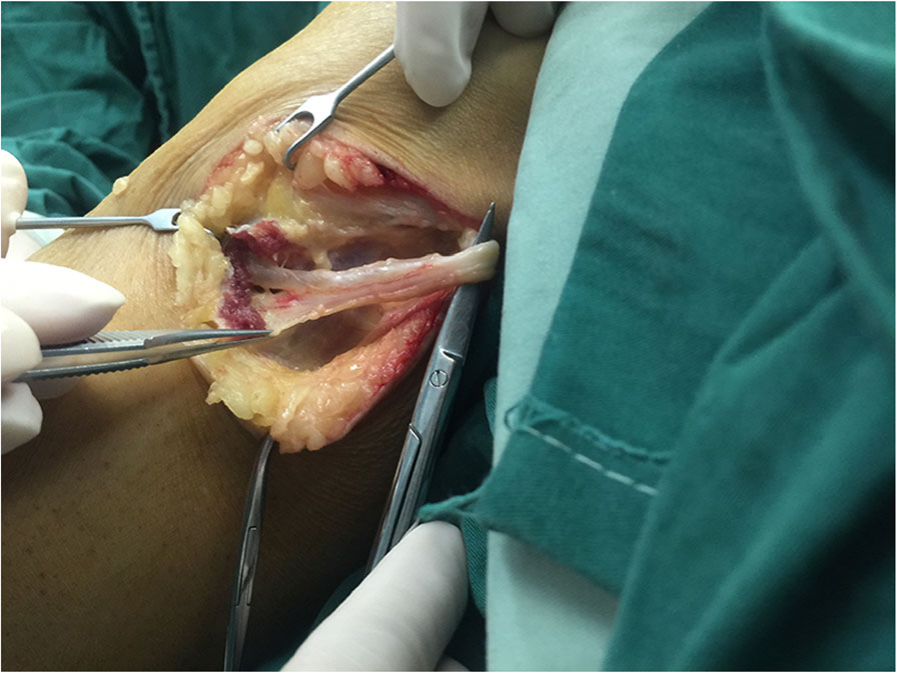
Cystic expansion was evident in the deep peroneal nerve

**Fig. 5:**
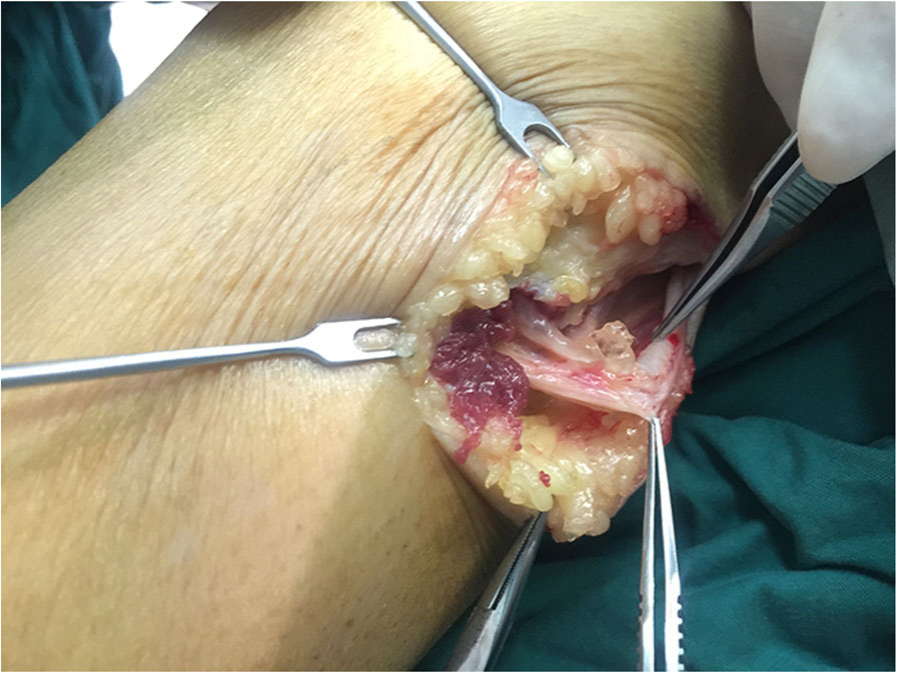
An abundant amount of mucoid material was evacuated from the incised intraneural ganglion cyst

**Fig. 6:**
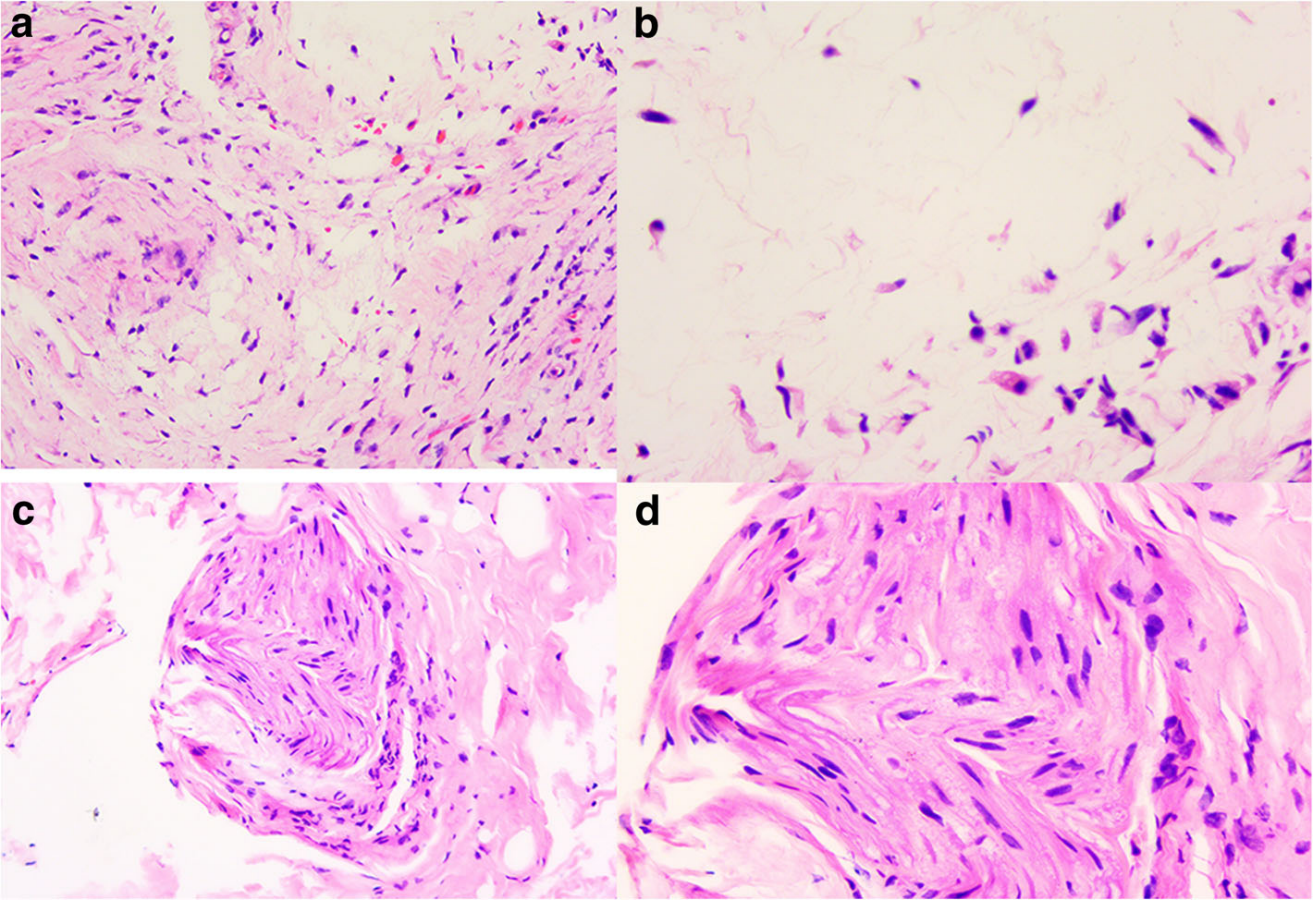
Pathology findings. **a** Mucoid degeneration in the small bit fibrous tissues of cystiform (20X 10). **b** Mucoid degeneration in small bit fibrous tissues of cystiform (40X 10). **c** Nerve tissue (20X 10). **d** Nerve tissue (40X 10)

We explored the common fibular nerve surgically. The common fibular nerve was identified and was traced to its bifurcation. The deep peroneal nerve was swollen. A fusiform cystic mass was located within the epineurium (Fig. 3). The cyst was found to track along the deep branches of the peroneal nerve (Fig. 4). A longitudinal incision was made on the cystic wall, mucoid material was evacuated from the cystic mass (Fig. 5), and part of the cystic wall and synovium were removed. The articular branch was not found. The surgical procedure was carried out with surgical loupes. Pathology showed a cystic wall structure with mucoid material, blood vessel scar fibrotic tissue, and neural tissue proliferation. From the operation and pathology report, the diagnosis was intraneural ganglion cyst with tissue hemorrhage and hydrosarca (Fig. 6). The patient was kept non-weight bearing for one month postoperatively with physiotherapy. She reported the return of sensation to the first and second toes three months after surgery and achieved a significant recovery. EMG investigations showed that the nerve conduction velocity improved three months later, the motor amplitude and denervation potential in the extensor hallucis longus (EHL) and tibialis anterior muscles improved. The muscle strength was fully restored relative to the contralateral leg. The patient was asymptomatic and able to return to her normal activities at one-year follow-up.

Written informed consent was obtained from the patient for publication of this case report and any accompanying images. Ethical approval was given by the medical ethics committee of the First Affiliated Hospital, College of Medicine, Zhejiang University.

## Discussion

The etiology of intraneural ganglion cysts is still controversial. In the past few years, evidence has been presented to support the theory of articular unification as mechanism [5]. Trauma has been suggested as another possible mechanism for the etiology of intraneural ganglion cyst [6]. The myxoid degeneration of connective tissue within the nerve forms the ganglion cyst after trauma. Mucoid degeneration of the epineurium causes the de novo cyst formation within the nerve sheath. Our case suggests that the intraneural ganglion cyst may have previously existed in some patients. Our patient had a history of trauma 12 days before her foot drop (the shortest time since trauma onset in the literature). The lesion may have been present at a subclinical level before the trauma. Local hemorrhage and tissue hydrosarca caused by high energy trauma decreased the space between nerve and cyst. So, the patient could have rapidly become symptomatic, in contrast to the Spinner’s mechanism [6].

A ganglion cyst is a rare cause of foot drop due to superior tibiofibular joint nerve compression. Both an extraneural or intraneural ganglion cyst can cause this symptom, which may need surgical treatment. Extraneural ganglion cysts derive also from the synovium, which is located separately from the epineurium of the peroneal nerve [7–9]. Muscle weakness [10], pain over the fibular head area [11], and a painful swelling [12] can be the initial symptoms of an intraneural ganglion cyst of the superior tibiofibular joint. One patient’s foot drop disappeared after physiotherapy, but after two months the symptom returned. The author of that report could not explain exactly what the mechanism was in that case [13]. Foot drop caused by ganglion cyst in superior tibiofibular joint is more rare in patients under 18 years of age [14].

Conservative treatment is not recommended, because nerve function would not fully recover because of the slowly enlarging cyst. The standard treatment is surgical excision of the ganglion and nerve decompression. However, post-surgical recurrence rate can be 30% [5], and surgery could present a high risk of complications including permanent nerve injury, vessel injury, and tissue infection. On the other hand, percutaneous intraneural cyst aspiration and injection of a corticosteroid has been proposed as a minimally invasive alternative to surgery for treating intraneural ganglion cysts [15]. However, the association between cyst recurrence and percutaneous aspiration was seen to be statistically significant, and potential complications include failure to disconnect the articular branch or address the joint [5].

Surgery can be performed before the onset of axonal injury if the diagnosis is made early [16, 17]. However, the intraneural ganglion cyst frequently cannot be identified by routine neurologic examination and EMG check before the operation. Ultrasound or MRI appears to be particularly important in these cases. In addition, the ganglion could appear as a cystic unenhanced lesion of mucoid density in CT [18]. Whether or not Ultrasound or MRI is a cost-effective screening method for nerve injury awaits further investigation.

## Conclusion

The concurrence of an intraneural ganglion cyst and trauma may increase damage to the nerve, although the cystis difficult to diagnose before an operation. Early diagnosis and early proactive interventions will usually be associated with good outcomes.

## Abbreviations

EHL: Extensor hallucis longus
EMG: Electromyography
ESR: Erythrocyte sedimentation rate
MRC: Medical Research Council
MRI: Magnetic resonance imaging
TA: Tibialis anterior muscle
